# Prenatal substance exposure and 5-year child protection outcomes among infants not reported at birth

**DOI:** 10.1371/journal.pone.0357388

**Published:** 2026-09-23

**Authors:** Julia Reddy, Davida M. Schiff, Anna Austin, Hendree E. Jones, Laura J. Faherty, Rebecca Rebbe, Anissa Vines, Emily Putnam-Hornstein

**Affiliations:** 1 University of North Carolina Chapel Hill School of Social Work, Chapel Hill, North Carolina, United States of America; 2 MassGeneral Hospital for Children, Harvard Medical School, Boston, Massachusetts, United States of America; 3 Department of Anesthesiology, University of North Carolina at Chapel Hill, Chapel Hill, North Carolina, United States of America; 4 Department of Obstetrics & Gynecology, University of North Carolina Chapel Hill, Chapel Hill, North Carolina, United States of America; 5 RAND, Boston, Massachusetts, United States of America; 6 MaineHealth, Portland, Maine, United States of America; 7 Tufts University, Boston, Massachusetts, United States of America; 8 University of North Carolina Chapel Hill Gillings School of Global Public Health, Chapel Hill, North Carolina, United States of America; University of South Florida, UNITED STATES OF AMERICA

## Abstract

Discretionary child protection system (CPS) reporting policies allow some substance-exposed infants to be discharged from the hospital without a maltreatment report, but it is unknown whether risk for later system involvement varies according to exposure status. We conducted a retrospective cohort analysis of CPS involvement in the first 5 years of life as predicted by prenatal substance exposure among California births in 2018. At 5 years, less than 14% of exposed children experienced CPS reports of maltreatment harm or substantiated CPS reports. Exposed children were more likely to be reported for maltreatment harm at 5 years (adjusted Risk Difference (aRD)=0.03, 95%CI: 0.02, 0.04), including a subgroup with only cannabis exposure (aRD = 0.03, 95%CI: 0.02, 0.04) compared with unexposed infants. Prenatally exposed children were more likely to experience a substantiated report within 5 years compared with those without exposure (aRD = 0.03, 95%CI: 0.02, 0.03). Findings suggest that families affected by prenatal substance exposure who do not have apparent safety concerns at birth may benefit from support to prevent future instances of system intervention or child maltreatment.

## Introduction

The child protection system (CPS) is the primary mechanism for responding to births of infants with prenatal substance exposure (IPSE) [1,2]. This reliance stems from evidence linking certain types of prenatal and parental substance use to increased risks of child maltreatment and child fatalities [3–7]. Increasingly, calls for a public health response to IPSE emphasize voluntary, preventive, and harm reductive services designed to support families affected by drug and alcohol use while preserving family cohesion [8–12].

Current federal statute, in the form of the amended Child Abuse Prevention and Treatment Act (CAPTA), requires states to develop a process to notify CPS about IPSE births, although CAPTA and supporting documentation are explicit that IPSE notifications need not take the form of maltreatment allegations [13,14]. States vary in their interpretation and policy responses to this law; very few states have instituted IPSE notification and response mechanisms separate from allegations of maltreatment [15].

California statute exemplifies a discretionary CPS reporting policy, empowering health care providers and other mandated reporters to assess the risk and safety of IPSE and determine whether a maltreatment allegation during the delivery hospitalization is warranted. The alternative to discretionary policies is universal reporting, in which all identified IPSE are reported to CPS after birth regardless of maltreatment risk. Research has demonstrated that discretionary policies encourage prenatal care seeking and limit CPS involvement to only those situations where infant safety is at risk [16]. However, the discretionary policy in California also exempts IPSE without safety concerns from the mandatory provision of a plan of safe care (POSC) and referrals to services [17,18].

While research has demonstrated associations between prenatal substance exposure and CPS reporting, these studies have included infants reported during the early neonatal period – when CPS contact may be related to policy adherence, rather than risk of maltreatment [19]. It is not known, at a population level, whether IPSE without safety concerns at birth are at higher risk of CPS contact during early childhood compared to infants discharged without CPS notification who do not have documented substance exposure. This question is particularly salient in a period where more states are legalizing cannabis use for medical and recreational purposes [20]. While the 2016 amendment of CAPTA includes exposures to legal substances, births with only cannabis exposures are consistently less likely to be reported to CPS than other substances [21,22] and it is not known whether cannabis-exposed births fail to receive adequate service referrals to protect against later CPS involvement [23].

This study examined incidence of CPS involvement in the first five years of life among a population of children born in California in 2018 who were not reported to CPS in the first 14 days, comparing those with and without documented prenatal substance exposure. We also assessed whether substance type (solely cannabis versus other or multiple substances) is associated with risk for later CPS contact compared with those without documented exposures.

## Methods

### Data

We used linked administrative data that included vital statistics birth records, maternal hospital discharge records, and CPS records. Vital statistical birth records were deterministically linked to maternal hospital discharge records. The resulting dataset was then probabilistically linked to CPS records using child, guardian(s), and residential identifiers using machine learning methods [24]. All analyses were based on deidentified records made available through data-sharing partnership agreements with county and state agencies. This secondary analysis falls under approvals from the University of North Carolina Institutional Review Board (UNC IRB #22–1100) and from the California Committee for the Protection of Human Subjects (CA State CPHS #13-10-1366). No study authors had access to identifiable information during or after data collection.

### Sample

We included all California live births from January 1 to December 31, 2018. We limited our analysis to first-born infants to eliminate any biasing effect of maternal CPS history, and to singleton births to ensure counting of family-level CPS referrals. We omitted births that were very low birth weight (weighed <1500 grams) and those that were born very preterm (<28 weeks) due to their likely longer length of hospital stay. Lastly, we omitted all infants who were reported to CPS within 14 days of birth, regardless of IPSE status, to maximize the likelihood that the infant was discharged from the hospital without a CPS report [25,26]. See S1 Fig for a depiction of our population.

### Measures

In this retrospective cohort study, we identified IPSE according to documentation in the maternal hospital discharge record of an International Classification of Diseases 10th revision, Clinical Modification (ICD-10-CM) code(s) related to drug or alcohol use disorder or a birth or pregnancy complicated by substance use. Included ICD-10-CM codes are based on prior research [27] and listed in S1 Table. Analytically, we compared infants with any substance exposure, cannabis-only exposure, and multi-/other substance exposure (i.e., substances other than cannabis or multiple substances) with infants with no documented substance exposure.

We examined outcomes of reports of maltreatment harm and any substantiated CPS reports. We defined a *report of maltreatment harm* as any CPS report, regardless of disposition, that carried an allegation type of physical abuse, severe neglect, caregiver incapacitation, or sexual abuse/exploitation. In California, the allegation type of caregiver incapacitation means significant parental inability to adequately care for the child and often indicates situations where parental substance use creates child harm or risk of harm [28]. We defined a *substantiated report* as any CPS report confirmed by the agency as substantiated maltreatment, regardless of allegation type, except those with an allegation type pertaining only to the maltreatment of a sibling. We measured outcomes as binary at 6 months, 1 year, 2 years, and 5 years following birth. Additionally, we measured each outcome in days to the first instance within 5 years of life.

Inclusion of covariates was guided by extant research [27,29–33]. Maternal race and ethnicity was self-reported on the birth records (White non-Hispanic; Black, non-Hispanic; all other single racial categorizations, non-Hispanic; two or more races, any Hispanic ethnicity; and any single racial categorization with Hispanic ethnicity) [34]. We also included maternal age at birth (<19; 20–24; 25–34; 35–55), maternal education (high school or less; some college or more), and paternity establishment based on demographic information of the father or second parent on the birth record (yes; no). Birth payment type for the delivery hospitalization was dichotomized as public (public health insurance) or private (private insurance or self-pay).

For maltreatment harm reporting, we adjusted for any prior CPS reports alleging general neglect or emotional abuse. For substantiated reports, we adjusted for prior allegations that were not substantiated [35]. Variable missingness was found to be in fewer than 1% of records.

### Analyses

Descriptive and bivariate analyses were conducted to determine the frequencies of covariates and dependent variables, and Chi square analyses were used to compare the distribution of these variables by level of prenatal substance exposure. We applied the Kaplan-Meier estimator to generate survival curves, plotted with time on the x-axis, from birth to five years, and unadjusted cumulative probabilities on the y-axis. We used a log-rank test to compare survival across substance exposure levels and pairwise log rank tests between cannabis-only and other/multiple substance exposure, with a Bonferroni correction to account for multiple comparisons.

We specified unadjusted and multivariable Poisson regressions, presented as risk ratios (RR) with 95% confidence intervals (CI), to compare associations between IPSE diagnosis at birth and CPS contact by age 6 months, 1 year, 2 years, and 5 years [36,37]. Robust sandwich variance estimation was used to correct for an overly wide confidence interval imposed by the Poisson distribution compared to a log binomial model. The ratio of both deviance and chi-square statistics to their degrees of freedom were below 1, confirming no evidence of overdispersion. Because measures of association that rely on ratios can be difficult to interpret or apply to clinical understanding, as they lack information about baseline prevalence, we also present adjusted risk differences (RD) derived from logistic regression models [38]. Placements in out of home care (foster care) prior to the first allegation of maltreatment harm were identified and sensitivity analyses were conducted excluding these cases to ensure that allegations of harm that occurred during or after an out of home placement did not bias our results. All analyses were conducted using Stata version 18.0.

### Limitations

This analysis has limitations. Diagnostic identification of prenatal substance exposure relies on provider screening, testing, and documentation, all of which may be affected by jurisdictional and hospital policies as well as norms of behavior and provider discretion. Similarly, prior research has exemplified inconsistencies, biases, and discrimination in CPS reporting [39].

This analysis had access only to the maternal hospital discharge record, and may have omitted substance exposed infants that were identified in the infant record only [40]. This may have resulted in some infants with prenatal substance exposure being included in the unexposed comparison group. However, this is likely to have had a conservative effect on our outcomes, given the known association between parental substance use and CPS involvement [1]. Our inability to access the infant hospital record also led to our use of a 14-day CPS reporting cutoff rather than the actual infant discharge date. Excluding very low birth weight and very preterm births increased the likelihood of alignment between the 14 day threshold and infant hospital discharge; however, it may be possible that we misclassified children who were reported following discharge but in the first two weeks as well as those who were reported after two weeks but still during their hospital stay. Omitted variables, including more nuanced measurements of economic positioning and other factors such as family violence, may explain some of the association between substance exposure and later CPS involvement.

## Results

In 2018 there were 160,725 births in California that met our inclusion criteria and that matched to a hospital discharge record (91% match rate) (S1 Fig). Just over 1% were identified as IPSE according to diagnoses on the maternal hospital discharge record (n = 1,800) and about 65% of those had cannabis as the only documented exposure (n = 1,168). A maltreatment harm report occurred in 0.5% of all births by 6 months, 0.9% by one year, 1.6% by 2 years, and 3.5% by five years. Among IPSE, 2.7% experienced a report of maltreatment harm at 6 months, 4.5% at 1 year, 6.7% at 2 years, and 13.2% at 5 years. At 6 months, 0.3% of all infants experienced a substantiated maltreatment report, 0.6% at 1 year, 1.2% at 2 years, and 2.1% at five years. Among IPSE, 1.8% experienced a substantiated report at 6 months, 3.6% at 1 year, 6.1% at 2 years, and 9.8% at 5 years (Table 1). Chi square analyses showed significant associations among all included dependent variables (Table 1) and covariates across the three-level substance exposure type (births with no documented exposure, cannabis-only, and other or multiple substance exposure) (Table 2).

**Table 1:** CPS outcomes by prenatal substance exposure category.

	Births not reported in first 14 days (N = 160,725)	No substance exposure (n-158,925; 98.9%)	Any substance exposure (n = 1,800; 1.1%)	Cannabis-only exposure (n = 1,168; 0.7%)	Other-/multi- substance exposure (n = 632; 0.4%)
**CPS Involvement**	**No. (%)**	**No. (%)**	**No. (%)**	**No. (%)**	**No. (%)**
Report of maltreatment harm					
6 months	758 (0.5)	709 (0.5)	49 (2.7)	29 (2.5)	20 (3.2)
1 year	1,473 (0.9)	1,392 (0.9)	81 (4.5)	53 (4.5)	28 (4.4)
2 years	2,569 (1.6)	2,448 (1.5)	121 (6.7)	84 (7.2)	37 (5.9)
5 years	5,639 (3.5)	5,402 (3.4)	237 (13.2)	161 (13.8)	76(12.0)
Substantiated report					
6 months	518 (0.3)	486 (0.3)	32 (1.8)	15 (1.3)	17 (2.7)
1 year	1,038 (0.7)	974 (0.6)	64 (3.6)	37 (3.2)	27 (4.3)
2 years	1,859 (1.2)	1,750 (1.1)	109 (6.1)	63 (5.4)	46 (7.3)
5 years	3,420 (2.1)	3,243 (2.0)	177 (9.8)	104 (8.9)	73 (11.6)

Note: Chi square tests compare the distribution of each dependent variable across substance exposure type groups. All Chi-square p values <0.001.

**Table 2:** Descriptive characteristics by prenatal substance exposure category.

	Births not reported in first 14 days (n = 160,725)	No substance exposure (n = 158,925; 98.9%)	Cannabis-only exposure (n = 1,168; 0.7%)	Other-/multi- substance exposure (n = 632; 0.4%)
	No. (%)	No. (%)	No. (%)	No. (%)
**Birth Payment**				
Public insurance	56,041 (34.9)	55,066 (34.6)	674 (57.7)	301 (47.6)
Private insurance or self-pay	104,512 (65.0)	103,695 (65.2)	491 (42.0)	326 (51.6)
**Maternal Race/Ethnicity**				
White, Non-Hispanic	46,066 (28.7)	45,450 (28.6)	345 (29.5)	271 (42.9)
Black, Non-Hispanic	7,073 (4.4)	6,820 (4.3)	206 (17.6)	47 (7.4)
AI/AN/A/H/PI/not listed, non-Hispanicª	30,858 (19.2)	30,768 (19.4)	52 (4.5)	38 (6.0)
Two or more races, any Hispanic status	6,338 (3.9)	6,172 (3.9)	128 (11.0)	38 (6.0)
Hispanic ethnicity, any single race	64,710 (40.3)	64,128 (40.4)	378 (32.4)	204 (32.3)
Not stated/ Unknown	5,680 (3.5)	5,587 (3.5)	59 (5.1)	34 (5.4)
**Maternal Age at Birth**				
<19 years	10,124 (6.3)	9,930 (6.2)	143 (12.2)	51 (8.1)
20-24 years	34,660 (21.6)	33,980 (21.4)	515 (44.1)	165 (26.1)
25-34 years	87,878 (54.7)	87,094 (54.8)	456 (39.0)	328 (51.9)
35 - 55 years	28,062 (17.5)	27,920 (17.6)	54 (4.6)	88 (13.9)
**Maternal Education**				
High school or less	54,560 (33.9)	53,523 (33.7)	725 (62.1)	312 (49.4)
Some college or more	106,165 (66.1)	105,402 (66.3)	443 (37.9)	320 (50.6)
**Paternity**				
Missing	10,567 (6.6)	10,254 (6.5)	171 (14.6)	142 (22.5)
Established	150,158 (93.4)	148,671 (93.5)	997 (85.4)	490 (77.5)

Note: Missing values excluded from percent calculation with the exception of race and ethnicity. Listed covariates were missing data in less than 1% of birth records. Chi-square tests compare the distribution of each dependent variable across substance exposure type groups. All Chi-square p values <0.001.

AI/ AN/ A/ H/ PI/ not listed, non-Hispanic = American Indian, Alaskan Native, Asian, Hawaiian, Pacific Islander, and all not listed racial categories, non-Hispanic.

**Fig 1:**
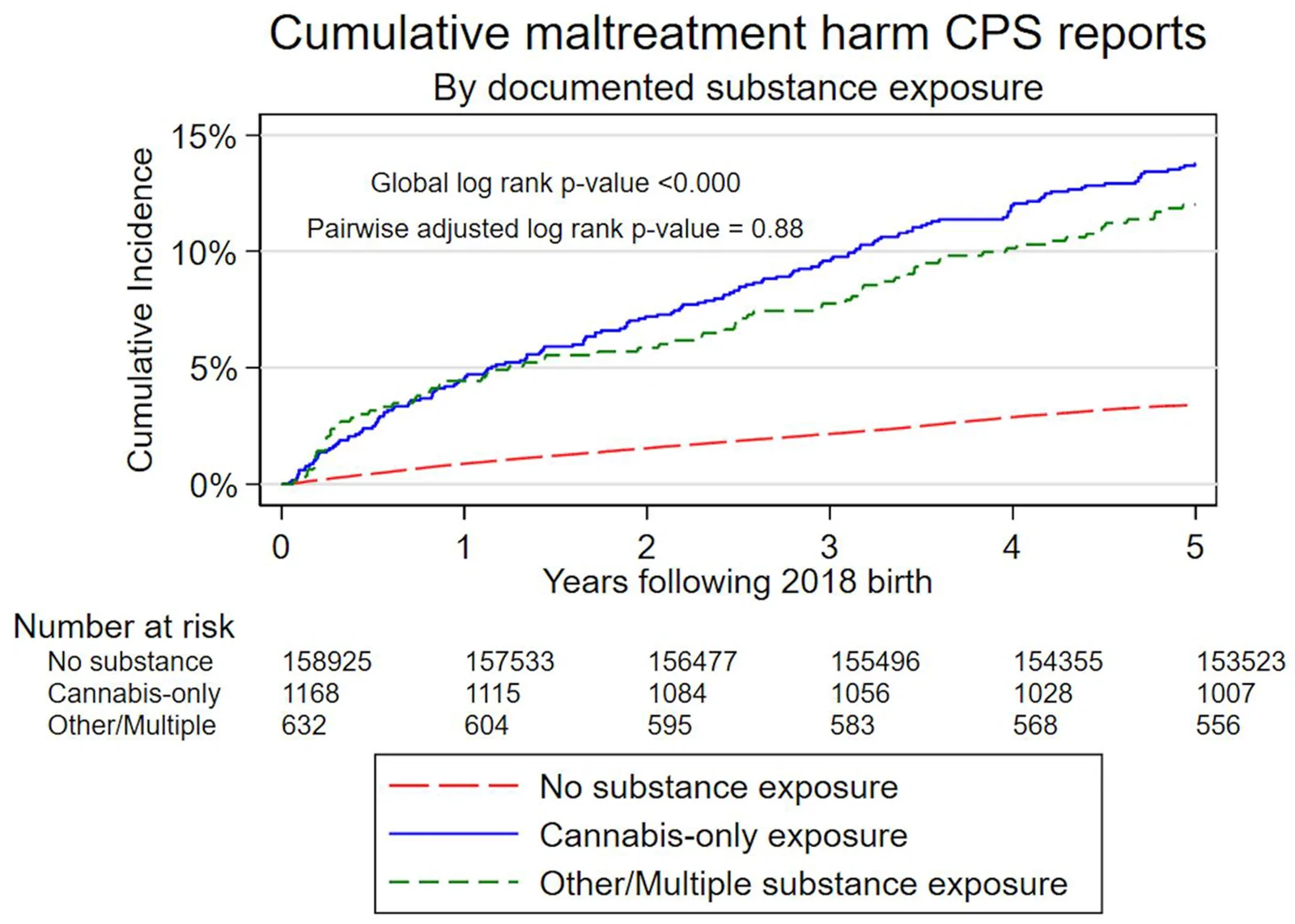
Cumulative probabilities of CPS outcomes by timepoint and prenatal substance exposure type. Report of maltreatment harm.

At six months, a greater percentage of births with multi-/other-substance exposure were reported for maltreatment harm (3.5%) compared to those with only cannabis exposure (2.5%) or no exposure (0.5%) but, at 2 years, births with only cannabis exposure had a higher incidence of maltreatment harm reporting (7.2%) than those with other substance exposure (5.9%) and no substance exposure (1.5%) (Table 1). Significant differences in survival probabilities were found among the three groups (log-rank Chi^2^ = 537.5, *p* < 0.000). However, pairwise comparisons with Bonferroni-adjusted p-values showed no significant differences between cannabis-only and multi-/other-substance exposed infants in the unadjusted cumulative probability of maltreatment harm reporting over 5 years (log-rank Chi^2^ = 1.1, *adjusted p* = 0.88) (Fig 1).

**Fig 2:**
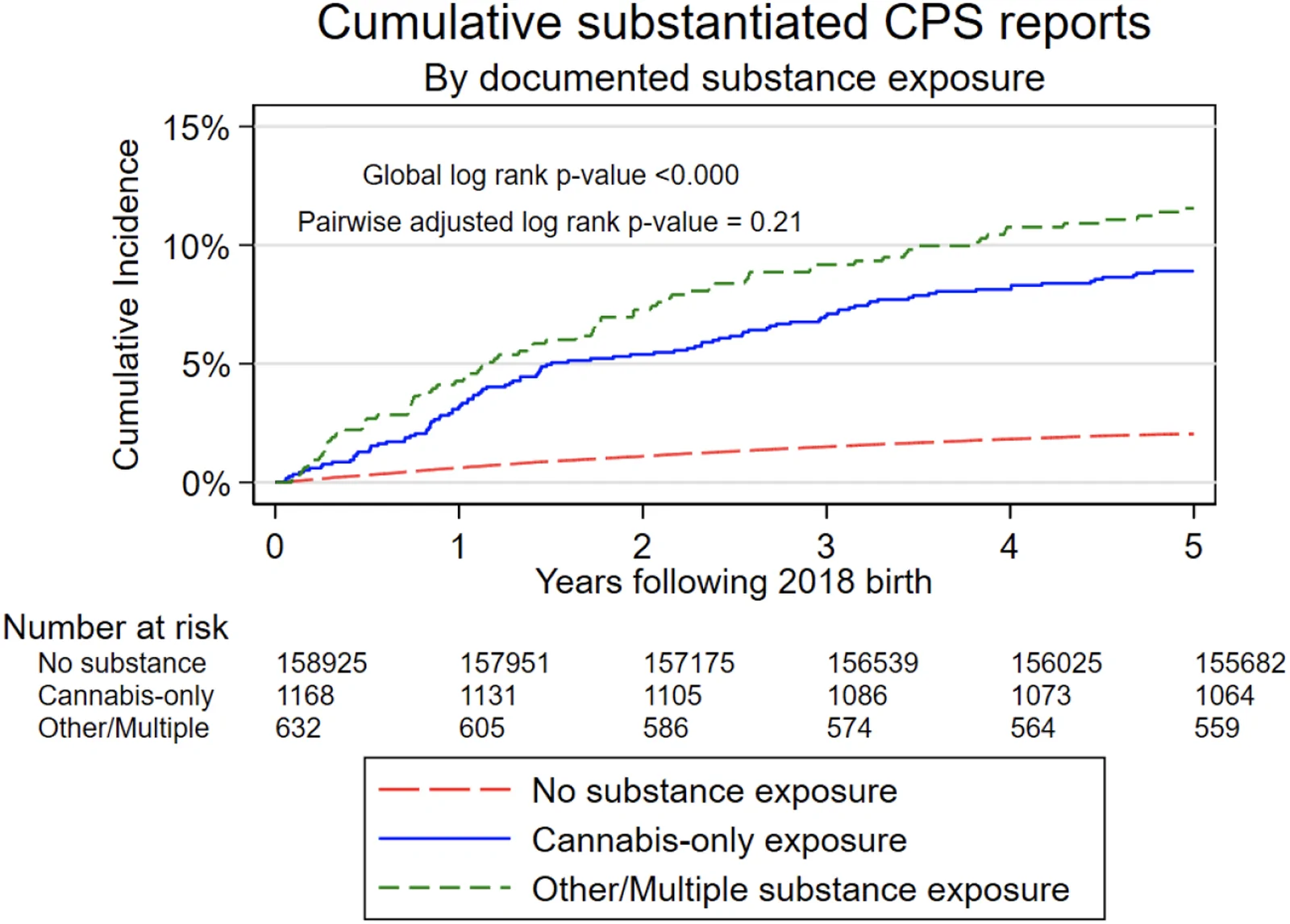
Cumulative probabilities of CPS outcomes by timepoint and prenatal substance exposure type. Substantiated CPS report.

At all four time points, incidence of a substantiated CPS report was higher among births exposed to other or multiple substances compared to those with cannabis-only and no exposure. However, adjusted pairwise comparisons reveal no significant difference in the unadjusted cumulative probability of a substantiated CPS report between cannabis-exposed infants and those exposed to other or multiple substances over 5 years (log-rank Chi^2^ = 3.3, *adjusted p* = 0.21) (Fig 2).

In multivariable models, IPSE were 1.9 (95% CI 1.6, 2.1) times as likely to be reported for maltreatment harm by age 5 compared to those with no documented exposure (Table 3).

**Table 3:** CPS Report of Maltreatment Harm: Unadjusted and Adjusted Models.

Model	Exposure	6 Months	1 Year	2 Years	5 Years
**Outcome prevalence**	**No substance exposure (n = 158,925)**	**0.5%**	**0.9%**	**1.5%**	**3.4%**
	Any substance exposure (n = 1,800)	2.7%	4.5%	6.7%	13.2%
	Cannabis-only (n = 1,168)	2.5%	4.5%	7.2%	13.8%
	Other/multi substance (n = 632)	3.2%	4.4%	5.9%	12.0%
**Unadjusted Risk Ratio**	**No substance exposure**	**1.00 (ref)**	**1.00 (ref)**	**1.00 (ref)**	**1.00 (ref)**
	Any substance exposure versus no exposure	6.1 (4.6-8.1)	5.1 (4.1-6.4)	4.4 (3.7-5.2)	3.9 (3.4-4.4)
	Cannabis-only versus no exposure	5.6 (3.9-8.0)	5.2 (4.0-6.8)	4.7 (3.8-5.8)	4.1 (3.5-4.7)
	Other/multi substance versus no exposure	7.1 (4.6-11.0)	5.1 (3.5-7.3)	3.8 (2.8-5.2)	3.5 (2.9-4.4)
**Adjusted Risk Ratio**	**No substance exposure**	**1.00 (ref)**	**1.00 (ref)**	**1.00 (ref)**	**1.00 (ref)**
	Any substance exposure versus no exposure	3.1 (2.3-4.2)	2.6 (2.0-3.2)	2.1 (1.8-2.6)	1.9 (1.6-2.1)
	Cannabis-only versus no exposure	2.6 (1.8-3.7)	2.3 (1.8-3.1)	2.1 (1.7-2.6)	1.8 (1.5-2.1)
	Other/multi substance versus no exposure	4.4 (2.8-7.0)	3.0 (2.1-4.4)	2.2 (1.6-3.0)	2.0 (1.6-2.5)
**Adjusted Risk Difference**	**No substance exposure**	**0.00 (ref)**	**0.00 (ref)**	**0.00 (ref)**	**0.00 (ref)**
	Any substance exposure versus no exposure	0.01 (0.006-0.014)	0.01 (0.01-0.02)	0.02 (0.01-0.03)	0.03 (0.02-0.03)
	Cannabis-only versus no exposure	0.007 (0.003-0.012)	0.012 (0.006-0.18)	0.01 (0.01-0.03)	0.03 (0.02-0.03)
	Other/multi substance versus no exposure	0.016 (0.007-0.024)	0.018 (0.008-0.03)	0.02 (0.01-0.03)	0.04 (0.02-0.05)

Cannabis-only exposure (aRR = 1.8, 95% CI 1.5, 2.1) and exposure to other or multiple substances (aRR = 2.0, 95% CI 1.6, 2.5) were both associated with an increased risk of a maltreatment harm report compared with no exposure. While the magnitude of the aRR varied somewhat over time, results were largely consistent for maltreatment harm by age 6 months, 1 year, and 2 years.

The adjusted risk difference of five-year maltreatment harm reporting was roughly 3 percentage points (95% CI 0.02, 0.04) higher among those with any substance exposure, cannabis-only exposures (aRD = 0.03; 95%CI = 0.02, 0.04), and other exposures (aRD = 0.04; 95%CI = 0.02, 0.05) compared to unexposed infants. The adjusted risk of five-year maltreatment harm reporting among infants with any exposure was 6.7%, cannabis-only exposure was 6.5%, and other substance exposure was 7.0%, compared to 3.4% for unexposed infants (Table 3).

Infants with any prenatal substance exposure were 2.2 (95% CI 1.9, 3.6) times as likely to be the subject of a substantiated maltreatment report at age 5 compared to those with no exposure. Cannabis-only exposure (aRR = 1.8; 95%CI = 1.5, 2.2) and other/multiple substance exposure (aRR = 3.3; 95%CI = 2.6, 4.0) were also associated with an increased risk of a substantiated CPS report compared with no exposure (Table 4). These results were fairly consistent across the four time points. The five-year adjusted risk of substantiated reporting among IPSE overall was 4.7%, including 3.8% among cannabis-exposed, and 6.8% among infants exposed to other/multiple substances. Compared to 2.1% among unexposed infants, this represented an adjusted risk difference of less than 5 percentage points (Table 4).

**Table 4:** Substantiated Maltreatment Report: Unadjusted and Adjusted Models.

Model	Exposure	6 Months	1 Year	2 Years	5 Years
**Outcome prevalence**	**No substance exposure (n = 158,925)**	**0.3%**	**0.6%**	**1.1%**	**2.0%**
	Any substance exposure (n = 1,800)	1.8%	3.6%	6.1%	9.8%
	Cannabis-only (n = 1,168)	1.3%	3.2%	5.4%	8.9%
	Other/multi substance (n = 632)	2.7%	4.3%	7.3%	11.6%
**Unadjusted Risk Ratio**	**No substance exposure**	**1.00 (ref)**	**1.00 (ref)**	**1.00 (ref)**	**1.00 (ref)**
	Any substance exposure versus no exposure	5.8 (4.1-8.3)	5.8 (4.5-7.3)	5.5 (4.6-6.6)	4.8 (4.2-5.6)
	Cannabis-only versus no exposure	4.2 (2.5-7.0)	5.2 (3.7-7.1)	4.9 (3.8-6.3)	4.4 (3.6-5.3)
	Other/multi substance versus no exposure	8.8 (5.5-14.2)	7.0 (4.8-10.1)	6.6 (5.0-8.8)	5.7 (4.5-7.0)
**Adjusted Risk Ratio**	**No substance exposure**	**1.00 (ref)**	**1.00 (ref)**	**1.00 (ref)**	**1.00 (ref)**
	Any substance exposure versus no exposure	2.7 (1.9-4.0)	2.6 (2.0-3.4)	2.5 (2.1-3.1)	2.2 (1.9-3.6)
	Cannabis-only versus no exposure	1.7 (1.0-3.0)	2.1 (1.5-3.0)	2.0 (1.6-2.6)	1.8 (1.5-2.2)
	Other/multi substance versus no exposure	5.1 (3.1-8.5)	3.9 (2.7-5.8)	3.7 (2.8-5.0)	3.3 (2.6-4.0)
**Adjusted Risk Difference**	**No substance exposure**	**0.00 (ref)**	**0.00 (ref)**	**0.00 (ref)**	**0.00 (ref)**
	Any substance exposure versus no exposure	0.006 (0.002-0.009)	0.011 (0.006-0.015)	0.02 (0.01-0.03)	0.03 (0.02-0.03)
	Cannabis-only versus no exposure	0.002 (−0.001-0.005)	0.007 (0.003-0.011)	0.01 (0.01-0.02)	0.02 (0.01-0.03)
	Other/multi substance versus no exposure	0.013 (0.005-0.021)	0.019 (0.009-0.028)	0.03 (0.02-0.04)	0.05 (0.03-0.06)

Results did not meaningfully change when infants with an out of home placement prior to a report of maltreatment harm (N = 102) were excluded from analyses (five-year aRR = 2.2, 95% CI 2.0, 2.5).

## Discussion

In this retrospective cohort analysis of infants born in California in 2018 who were not reported to CPS in the first two weeks of life, we found higher risk of CPS outcomes among those with prenatal substance exposure compared with infants without documented exposure that persisted with adjustment for confounding factors, over time, and across modeling strategies. Additionally, the unadjusted cumulative probability of being reported for maltreatment harm or having a substantiated CPS report in five years did not significantly differ between infants with documented exposure to only cannabis and those with documented exposure to other or multiple substances. Yet, when demographic and prior CPS contact measures were adjusted for, differences in risk of substantiated reporting according to substance exposure types emerged. Overall, prevalence of CPS outcomes among IPSE was below 14% over five years, and the adjusted risk difference between exposed to unexposed infants was less than five percentage points.

Under the current CAPTA authorization, there is ambiguity around which IPSE populations should be reported to the federal government through state child protection systems. In 2021, 49 states reported fewer than 49,000 IPSE, representing less than 1.3% of the birth population [41] – significantly less than the 13.9% of pregnant people self-reporting past month alcohol or illicit drug use (including cannabis) [42]. In California, IPSE are defined only as those substance exposed newborns who also have safety concerns justifying a report to CPS; only those families are mandated to receive a POSC. This exempts infants without safety concerns from potentially invasive or intimidating CPS intervention, but it also excludes them from mandated safety planning and service referrals. Our finding of nearly twice the adjusted risk of CPS outcomes among substance exposed newborns compared to those without substance exposure among a population of infants without a neonatal CPS report indicates that prevention services may be needed to prevent future CPS involvement or child harm. Alternate pathways to supportive family services outside of CPS intervention are essential parts of a comprehensive public health response to prenatal substance exposure with the potential to mitigate later risk [43].

Prior associations have demonstrated decreased risk of CPS reporting [22,27] and out of home placements [44] among children with cannabis exposure compared to other substance exposures. An important distinction in the current study is the omission of all infants who were reported prior to hospital discharge, or in the first 14 days of life. Our findings suggest that, among IPSE determined to be low risk by the birth hospital, those with documented cannabis exposure experience elevated CPS involvement over time compared to infants with no prenatal substance exposure. The increasing prevalence and perceived innocuity of prenatal cannabis use requires a targeted programmatic response to address the needs of exposed infants, whether related to sequelae of exposure or corollary family challenges [45–47].

While our analyses found no difference between unadjusted risk ratios of maltreatment harm and substantiated reporting over five years among infants with cannabis-only compared to those with other/multi-substance exposures, adjustment for demographic and birth characteristics created measurable differences across substance types for the outcome of substantiated reporting. Put another way, the adjusted risk of maltreatment harm reporting is not different according to substance type while the risk of substantiated reporting is. This may speak to the association between continued parental use of substances other than cannabis and substantiated CPS reporting in early childhood. Prenatal substance use is a reliable indicator of parental substance use, which is strongly associated with CPS intervention [48,49]. Outside of CPS referrals at the birth event, families affected by prenatal substance use should be assessed for risk of parental use and interventions supporting recovery and parenting competence should be readily available if we are to avoid later CPS involvement.

After five years, 13.2% of IPSE not reported to CPS immediately after birth experienced a report of maltreatment harm and 9.8% experienced a substantiated CPS report. That is, in a lower risk sample of infants that were deemed to not need reporting at delivery, 86% had neither form of CPS involvement in the first 5 years of life. While measured interactions with CPS are higher for IPSE than unexposed infants, the adjusted risk difference for either CPS outcome comparing IPSE with non-IPSE was below 5 percentage points. Importantly, the risk difference was nearly 10 percentage points lower after adjustment for socioeconomic and demographic factors and other CPS interactions. This suggests that, rather than prenatal substance exposure alone, a constellation of intersecting risk factors may contribute to elevated CPS involvement over early childhood and should be targeted by prevention support program design. As more jurisdictions attempt to institute harm reductive and family-centered responses to perinatal substance use, discretionary reporting alongside universal service provision should be prioritized.

## Supporting information

S1 Fig Derivation of study population. (DOCX)

S1 Table Classification of Prenatal Substance Use by ICD-CM-10 Codes. (DOCX)
