# Supplementary material for: Prenatal substance exposure and 5-year child protection outcomes among infants not reported at birth
Source: PLoS One. 2026 Sep 23;21(9):e0357388. doi: 10.1371/journal.pone.0357388 (PMC13600510; doi:10.1371/journal.pone.0357388)
Supplement: Supplementary file 2 [file pone.0357388.s002.docx]

**S1 Table. Classification of Prenatal Substance Use by ICD-CM-10 Codes**

|  | ICD-CM-10 Code |
| --- | --- |
| Alcohol related disorders | F10  (except F10.11, F10.21, F10.91) |
| Opioid related disorders | F11  (except F11.11, F11.21, F11.91) |
| Cannabis related disorders | F12  (except F12.11, F12.21, F12.91) |
| Sedative, hypnotic, or anxiolytic related disorders | F13  (except F13.11, F13.21, F13.91) |
| Cocaine related disorders | F14  (except F14.11, F14.21, F14.91) |
| Other stimulant related disorders | F15  (except F15.11, F15.21, F15.91) |
| Hallucinogen related disorders | F16  (except F16.11, F16.21, F16.91) |
| Inhalant related disorders | F18  (except F18.11, F18.21, F18.91) |
| Other psychoactive substance related disorders | F19  (except F19.11, F19.21, F19.91) |
| Alcohol use complicating pregnancy, childbirth, and the puerperium | O99.31 |
| Drug use complicating pregnancy, childbirth, and the puerperium | O99.32 |
| Maternal care for (suspected) damage to fetus from alcohol | O35.4 |
| Maternal care for (suspected) damage to fetus by drugs | O35.5 |
