# Supplementary material for: Prenatal substance exposure and 5-year child protection outcomes among infants not reported at birth
Source: PLoS One. 2026 Sep 23;21(9):e0357388. doi: 10.1371/journal.pone.0357388 (PMC13600510; doi:10.1371/journal.pone.0357388)
Supplement: Supplementary file 3 [file pone.0357388.s003.docx]

Article Review

I was asked to review the article entitled “Prenatal Substance Exposure and 5-Year Child Protection Outcomes Among Infants Not Reported at Birth”, which was submitted to PLOS ONE. The article presents a retrospective cohort study using existing data from linked administrative data that included vital statistics birth records, maternal hospital discharge records, and CPS records. The topic is relevant to the readership of PLOS ONE since prenatal substance use is currently a major public health problem in the US and a frequent cause of child welfare involvement. The study has many strengths including the focus on discretionary policies in California, longitudinal design and 5 years of follow up data analyzed. Just minor changes are needed. Details of my assessment are below.

Title and abstract: The authors must indicate the study design (retrospective cohort study) either in the abstract or in the title. Currently, the abstract only indicates “cohort analysis”, which is insufficient.

Background: The scientific background and rationale for the investigation was explained clearly. The specific objectives were stated. No change recommended.

Methods: Indicate the study design as well at the beginning of the methods section in compliance with STROBE guidelines for observational studies. Since the study is a cohort study that uses data already collected in the past, the design is a retrospective cohort study.

Although authors cited a publication with the details of the record linkage, but a very brief description is still needed. For instance, it is necessary they declare that it was a probabilistic record linkage using machine learning rather than deterministic and also they can report the accuracy of the linkage. The cited publication compares three methods (three record linkage solutions: PRLF, ChoiceMaker, and Link Plus) and the reader is uncertain as to which of those was the one used in this study.

Results: In paragraph 1 of the results section, the authors stated: “A maltreatment harm report occurred in 0.5% of all births by 6 months, 0.9% by one year, 1.6% by 2 years, and 3.5% by five years. Among IPSE, 2.7% experienced a report of maltreatment harm at 6 months, 4.5% at 1 year, 6.7% at 2 years, and 13.2% at 5 years.” Table 1 was cited as the source, but Table 1 presents percentages for cannabis and other substances instead of all IPSE. Correct the issue in the table 1 by adding a column for total IPSE, or change the narrative to match what is the table (e.g., by comparing “not substance exposed” to “cannabis” and “other substances” group.

Analysis: The techniques used were described (Chi-square, Kaplan Meier, Poisson regression). However, a brief description of model diagnostics and goodness of fit indices is needed if available. For instance, for Poisson regression, the methods need to indicate if the following were consulted: dispersion statistic to detect overdispersion; model adequacy measures (such as deviance, Chi-square); if models were compared (AIC, BIC), and Pseudo-R square.

Also, please indicate how missing values were handled.

Conclusions: These were based on study findings and limitations were addressed.
