# Supplementary material for: Prenatal substance exposure and 5-year child protection outcomes among infants not reported at birth
Source: PLoS One. 2026 Sep 23;21(9):e0357388. doi: 10.1371/journal.pone.0357388 (PMC13600510; doi:10.1371/journal.pone.0357388)
Supplement: Supplementary file 4 [file pone.0357388.s001.docx]

**S1 Fig. Derivation of study population**
