# Supplementary material for: Prenatal substance exposure and 5-year child protection outcomes among infants not reported at birth
Source: PLoS One. 2026 Sep 23;21(9):e0357388. doi: 10.1371/journal.pone.0357388 (PMC13600510; doi:10.1371/journal.pone.0357388)
Supplement: Supplementary file 5 [file pone.0357388.s005.docx]

**Response to Reviewers:** PONE-D-25-68859

Prenatal Substance Exposure and 5-Year Child Protection Outcomes Among Infants Not Reported at Birth

April 21, 2026

Thank you very much for taking the time to thoughtfully review our manuscript submission. We have responded to each of your edits and comments below. We look forward to a review of this revision.

| Reviewer Comment | Author Response | Location of Revision |
| --- | --- | --- |
| 1. Please ensure that your manuscript meets PLOS ONE's style requirements, including those for file naming. The PLOS ONE style templates can be found at  [https://journals.plos.org/plosone/s/file?id=wjVg/PLOSOne_formatting_sample_main_body.pdf](https://track.editorialmanager.com/CL0/https:%2F%2Fjournals.plos.org%2Fplosone%2Fs%2Ffile%3Fid=wjVg%2FPLOSOne_formatting_sample_main_body.pdf/1/010f019d430c4a62-efd82747-6ce9-41c6-95ea-0d2696eae8e5-000000/-nWtSParAR9TOstLAfEFqGMYOLw8tZrViEjFeujgcuw=256) and [https://journals.plos.org/plosone/s/file?id=ba62/PLOSOne_formatting_sample_title_authors_affiliations.pdf](https://track.editorialmanager.com/CL0/https:%2F%2Fjournals.plos.org%2Fplosone%2Fs%2Ffile%3Fid=ba62%2FPLOSOne_formatting_sample_title_authors_affiliations.pdf/1/010f019d430c4a62-efd82747-6ce9-41c6-95ea-0d2696eae8e5-000000/I8WoGjVtqh2zMUM0rZR3OQdY8oWGnocc9ghBUqvxS64=256) | Thank you – we have reviewed these templates and ensured that our manuscript complies with the style requirements. | Throughout (revisions to Title Page) |
| 2. Thank you for stating in your Funding Statement:  [JR was supported by the National Institute on Drug Abuse of the National Institutes of Health under award number F31DA057756.The NIH played no role in the study design, data collection, analysis, preparation, nor decision to publish this manuscript.  RR was supported by the Centers for Disease Control and Prevention, under award number K01CE003548. The CDC played no role in the study design, data collection, analysis, preparation, nor decision to publish this manuscript.  LF was supported by the National Institute of Mental Health of the National Institutes of Health under award number R01MH138475.The NIH played no role in the study design, data collection, analysis, preparation, nor decision to publish this manuscript.].  Please provide an amended statement that declares *all* the funding or sources of support (whether external or internal to your organization) received during this study, as detailed online in our guide for authors at <http://journals.plos.org/plosone/s/submit-now>.  Please also include the statement “There was no additional external funding received for this study.” in your updated Funding Statement.  Please include your amended Funding Statement within your cover letter. We will change the online submission form on your behalf. | Thank you; we have amended the funding statement and relocated it to the cover letter. | COVER LETTER |
| 3. We note that you have indicated that there are restrictions to data sharing for this study. For studies involving human research participant data or other sensitive data, we encourage authors to share de-identified or anonymized data. However, when data cannot be publicly shared for ethical reasons, we allow authors to make their data sets available upon request. For information on unacceptable data access restrictions, please see <http://journals.plos.org/plosone/s/data-availability#loc-unacceptable-data-access-restrictions>.  Before we proceed with your manuscript, please address the following prompts:  a) If there are ethical or legal restrictions on sharing a de-identified data set, please explain them in detail (e.g., data contain potentially identifying or sensitive patient information, data are owned by a third-party organization, etc.) and who has imposed them (e.g., a Research Ethics Committee or Institutional Review Board, etc.). Please also provide contact information for a data access committee, ethics committee, or other institutional body to which data requests may be sent.  b) If there are no restrictions, please upload the minimal anonymized data set necessary to replicate your study findings to a stable, public repository and provide us with the relevant URLs, DOIs, or accession numbers. Please see <http://www.bmj.com/content/340/bmj.c181.long> for guidelines on how to de-identify and prepare clinical data for publication. For a list of recommended repositories, please see <https://journals.plos.org/plosone/s/recommended-repositories>. You also have the option of uploading the data as Supporting Information files, but we would recommend depositing data directly to a data repository if possible.  Please update your Data Availability statement in the submission form accordingly. | Thank you. Yes, the data are held by public agencies and permitted for use through restrictive data use agreements that are not transferrable. We have amended the data availability statement and included the contact information of the ethics review body. | SUBMISSION FORM and manuscript Line 84 |
| 4. Please ensure that you include a title page within your main document. You should list all authors and all affiliations as per our author instructions and clearly indicate the corresponding author. | Thank you – we have moved our title page to the manuscript file. | LINES 1 – 23 |
| 5. Please include your full ethics statement in the ‘Methods’ section of your manuscript file. In your statement, please include the full name of the IRB or ethics committee who approved or waived your study, as well as whether or not you obtained informed written or verbal consent. If consent was waived for your study, please include this information in your statement as well. | Thank you – we have added this information to the manuscript. | LINES 81-87 |
| **Reviewer 1:** P205 line 220, there is a space between 2.2_ ), and P234 line 252 “populations” is misspelled | Thank you for these edits; changes have been made. | LINE 246; LINE 289 |
| **Reviewer 2:** |  |  |
| Title and abstract: The authors should indicate the study design (retrospective cohort study) either in the abstract or in the title. Currently, the abstract only indicates “cohort analysis”, which is insufficient. | Thank you; we have added this language to the abstract, methods, and discussion sections. | LINE 28 |
| Methods: Indicate the study design as well at the beginning of the methods section in compliance with STROBE guidelines for observational studies (Best practice: <https://www.strobe-statement.org/>). Since the study is a cohort study that uses data already collected in the past, the design is a retrospective cohort study. | Yes, we have added this language | LINES 98 & 267 |
| Although authors cited a publication with the details of the record linkage, but a very brief description is still needed. For instance, it is necessary they declare that it was a probabilistic record linkage using machine learning rather than deterministic and also they can report the accuracy of the linkage. The cited publication compares three methods (three record linkage solutions: PRLF, ChoiceMaker, and Link Plus) and the reader is uncertain as to which of those was the one used in this study. | Thank you – this was added | LINES 78 - 82 |
| Results: In paragraph 1 of the results section, the authors stated: “A maltreatment harm report occurred in 0.5% of all births by 6 months, 0.9% by one year, 1.6% by 2 years, and 3.5% by five years. Among IPSE, 2.7% experienced a report of maltreatment harm at 6 months, 4.5% at 1 year, 6.7% at 2 years, and 13.2% at 5 years.” Table 1 was cited as the source, but Table 1 presents percentages for cannabis and other substances instead of all IPSE. Correct the issue in the table 1 by adding a column for total IPSE, or change the narrative to match what is the table (e.g., by comparing “not substance exposed” to “cannabis” and “other substances” group. | Ah – thank you. I have recreated the table with an added “any substance exposure” column. | LINE 189 |
| Analysis: The techniques used were described (Chi-square, Kaplan Meier, Poisson regression). However, a brief description of model diagnostics and goodness of fit indices is needed if available. For instance, for Poisson regression, the methods need to indicate if the following were consulted: dispersion statistic to detect overdispersion; model adequacy measures (such as deviance, Chi-square); if models were compared (AIC, BIC), and Pseudo-R square. | Information about robust variance estimators and model adjustment decisions were added to the Analysis section. | LINES 145-149 |
| Please indicate how missing values were handled. | The handling of missingness was recorded in the Table Notes, and the volume of missingness has been added to the Measures section. | LINE 125, 201 |
