# Supplementary material for: Prenatal substance exposure and 5-year child protection outcomes among infants not reported at birth
Source: PLoS One. 2026 Sep 23;21(9):e0357388. doi: 10.1371/journal.pone.0357388 (PMC13600510; doi:10.1371/journal.pone.0357388)
Supplement: Peer review history

# Peer review: Decision Letter 0

**DOI:** 10.1371/journal.pone.0357388.r001

**Authors:** Abraham Salinas-Miranda

31 Mar 2026

Dear Dr. Reddy,

Thank you for submitting your manuscript to PLOS ONE. After careful consideration, we feel that it has merit but does not fully meet PLOS ONE’s publication criteria as it currently stands. Therefore, we invite you to submit a revised version of the manuscript that addresses the points raised during the review process.

Please submit your revised manuscript by May 15 2026 11:59PM. If you will need more time than this to complete your revisions, please reply to this message or contact the journal office at plosone@plos.org. When you're ready to submit your revision, log on to https://www.editorialmanager.com/pone/ and select the 'Submissions Needing Revision' folder to locate your manuscript file.

- A letter that responds to each point raised by the academic editor and reviewer(s). You should upload this letter as a separate file labeled 'Response to Reviewers'.
- A marked-up copy of your manuscript that highlights changes made to the original version. You should upload this as a separate file labeled 'Revised Manuscript with Track Changes'.
- An unmarked version of your revised paper without tracked changes. You should upload this as a separate file labeled 'Manuscript'.

If applicable, we recommend that you deposit your laboratory protocols in protocols.io to enhance the reproducibility of your results. Protocols.io assigns your protocol its own identifier (DOI) so that it can be cited independently in the future. For instructions see: https://journals.plos.org/plosone/s/submission-guidelines#loc-laboratory-protocols. Additionally, PLOS ONE offers an option for publishing peer-reviewed Lab Protocol articles, which describe protocols hosted on protocols.io. Read more information on sharing protocols at https://plos.org/protocols?utm_medium=editorial-email&utm_source=authorletters&utm_campaign=protocols.

We look forward to receiving your revised manuscript.

Kind regards,

Abraham Salinas-Miranda, MD, PhD

Academic Editor

PLOS One

Journal Requirements:

When submitting your revision, we need you to address these additional requirements.

1. Please ensure that your manuscript meets PLOS ONE's style requirements, including those for file naming. The PLOS ONE style templates can be found at

https://journals.plos.org/plosone/s/file?id=wjVg/PLOSOne_formatting_sample_main_body.pdf and https://journals.plos.org/plosone/s/file?id=ba62/PLOSOne_formatting_sample_title_authors_affiliations.pdf

2. Thank you for stating in your Funding Statement:

[JR was supported by the National Institute on Drug Abuse of the National Institutes of Health under award number F31DA057756.The NIH played no role in the study design, data collection, analysis, preparation, nor decision to publish this manuscript.

RR was supported by the Centers for Disease Control and Prevention, under award number K01CE003548. The CDC played no role in the study design, data collection, analysis, preparation, nor decision to publish this manuscript.

LF was supported by the National Institute of Mental Health of the National Institutes of Health under award number R01MH138475.The NIH played no role in the study design, data collection, analysis, preparation, nor decision to publish this manuscript.].

Please provide an amended statement that declares *all* the funding or sources of support (whether external or internal to your organization) received during this study, as detailed online in our guide for authors at http://journals.plos.org/plosone/s/submit-now.  Please also include the statement “There was no additional external funding received for this study.” in your updated Funding Statement.

Please include your amended Funding Statement within your cover letter. We will change the online submission form on your behalf.

3. We note that you have indicated that there are restrictions to data sharing for this study. For studies involving human research participant data or other sensitive data, we encourage authors to share de-identified or anonymized data. However, when data cannot be publicly shared for ethical reasons, we allow authors to make their data sets available upon request. For information on unacceptable data access restrictions, please see http://journals.plos.org/plosone/s/data-availability#loc-unacceptable-data-access-restrictions.

Before we proceed with your manuscript, please address the following prompts:

a) If there are ethical or legal restrictions on sharing a de-identified data set, please explain them in detail (e.g., data contain potentially identifying or sensitive patient information, data are owned by a third-party organization, etc.) and who has imposed them (e.g., a Research Ethics Committee or Institutional Review Board, etc.). Please also provide contact information for a data access committee, ethics committee, or other institutional body to which data requests may be sent.

b) If there are no restrictions, please upload the minimal anonymized data set necessary to replicate your study findings to a stable, public repository and provide us with the relevant URLs, DOIs, or accession numbers. Please see http://www.bmj.com/content/340/bmj.c181.long for guidelines on how to de-identify and prepare clinical data for publication. For a list of recommended repositories, please see https://journals.plos.org/plosone/s/recommended-repositories. You also have the option of uploading the data as Supporting Information files, but we would recommend depositing data directly to a data repository if possible.

Please update your Data Availability statement in the submission form accordingly.

4. Please ensure that you include a title page within your main document. You should list all authors and all affiliations as per our author instructions and clearly indicate the corresponding author.

5. Please include your full ethics statement in the ‘Methods’ section of your manuscript file. In your statement, please include the full name of the IRB or ethics committee who approved or waived your study, as well as whether or not you obtained informed written or verbal consent. If consent was waived for your study, please include this information in your statement as well.

If the reviewer comments include a recommendation to cite specific previously published works, please review and evaluate these publications to determine whether they are relevant and should be cited. There is no requirement to cite these works unless the editor has indicated otherwise.

Please review your reference list to ensure that it is complete and correct. If you have cited papers that have been retracted, please include the rationale for doing so in the manuscript text, or remove these references and replace them with relevant current references. Any changes to the reference list should be mentioned in the rebuttal letter that accompanies your revised manuscript. If you need to cite a retracted article, indicate the article’s retracted status in the References list and also include a citation and full reference for the retraction notice.

Additional Editor Comments:

This paper was reviewed by an independent reviewer and this academic editor (serving as reviewer). The paper has value to the readership of PLOS ONE as there are not many studies assessing the risk of substantiated child maltreatment among children exposed to substance prenatally. The data linkage of multiple sources, the use of ICD 10 codes, and the longitudinal designs are all study strengths. Minor issues were identified that require your attention.

[Note: HTML markup is below. Please do not edit.]

Reviewers' comments:

Reviewer's Responses to Questions

**Comments to the Author**

1. Is the manuscript technically sound, and do the data support the conclusions?

Reviewer #1: Yes

Reviewer #2: Yes

**********

2. Has the statistical analysis been performed appropriately and rigorously?

Reviewer #1: Yes

Reviewer #2: Yes

**********

3. Have the authors made all data underlying the findings in their manuscript fully available?

The PLOS Data policy

Reviewer #1: No

Reviewer #2: Yes

**********

4. Is the manuscript presented in an intelligible fashion and written in standard English?

Reviewer #1: Yes

Reviewer #2: Yes

**********

Reviewer #1: This was a novel study conducted in California to examine the comparative risk of reported and substantiated CPS involvement for families of infants exposed to no substances, cannabis only, or other illicit substances, but not reported at birth for substance exposure due to discretionary reporting policies.

The study was well described and analysis appropriate. A major strength of the study is the longitudinal design (through 5 years postpartum) and risk comparison for cannabis versus other substances. The results are compelling, with important implications for other jurisdictions that are considering discretionary CPS reporting policies for substance exposure at birth. Finally, the conclusions are critical - reinforcing the importance of universal service provision to address intersecting risk factors when substance exposure at birth is present. Small editorial notes: P205 line 220, there is a space between 2.2_ ), and P234 line 252 “populations” is misspelled

Reviewer #2: Details of review are attached. I was asked to review the article entitled “Prenatal Substance Exposure and 5-Year Child Protection Outcomes Among Infants Not Reported at Birth”, which was submitted to PLOS ONE. The article presents a retrospective cohort study using existing data from linked administrative data that included vital statistics birth records, maternal hospital discharge records, and CPS records. The topic is relevant to the readership of PLOS ONE since prenatal substance use is currently a major public health problem in the US and a frequent cause of child welfare involvement. The study has many strengths including the focus on discretionary policies in California, longitudinal design and 5 years of follow up data analyzed. Just minor changes are needed. Details of my assessment are below.

Title and abstract: The authors should indicate the study design (retrospective cohort study) either in the abstract or in the title. Currently, the abstract only indicates “cohort analysis”, which is insufficient.

Background: The scientific background and rationale for the investigation was explained clearly. The specific objectives were stated. No change recommended.

Methods: Indicate the study design as well at the beginning of the methods section in compliance with STROBE guidelines for observational studies (Best practice: https://www.strobe-statement.org/). Since the study is a cohort study that uses data already collected in the past, the design is a retrospective cohort study.

Although authors cited a publication with the details of the record linkage, but a very brief description is still needed. For instance, it is necessary they declare that it was a probabilistic record linkage using machine learning rather than deterministic and also they can report the accuracy of the linkage. The cited publication compares three methods (three record linkage solutions: PRLF, ChoiceMaker, and Link Plus) and the reader is uncertain as to which of those was the one used in this study.

Results: In paragraph 1 of the results section, the authors stated: “A maltreatment harm report occurred in 0.5% of all births by 6 months, 0.9% by one year, 1.6% by 2 years, and 3.5% by five years. Among IPSE, 2.7% experienced a report of maltreatment harm at 6 months, 4.5% at 1 year, 6.7% at 2 years, and 13.2% at 5 years.” Table 1 was cited as the source, but Table 1 presents percentages for cannabis and other substances instead of all IPSE. Correct the issue in the table 1 by adding a column for total IPSE, or change the narrative to match what is the table (e.g., by comparing “not substance exposed” to “cannabis” and “other substances” group.

Analysis: The techniques used were described (Chi-square, Kaplan Meier, Poisson regression). However, a brief description of model diagnostics and goodness of fit indices is needed if available. For instance, for Poisson regression, the methods need to indicate if the following were consulted: dispersion statistic to detect overdispersion; model adequacy measures (such as deviance, Chi-square); if models were compared (AIC, BIC), and Pseudo-R square.

Also, please indicate how missing values were handled.

Conclusions: These were based on study findings and limitations were addressed. Kudos!

**********

what does this mean?). If published, this will include your full peer review and any attached files.

If you choose “no”, your identity will remain anonymous but your review may still be made public.

**Do you want your identity to be public for this peer review?**  For information about this choice, including consent withdrawal, please see our Privacy Policy

Reviewer #1: **Yes:**  Jennifer Marshall

Reviewer #2: **Yes:**  Abraham Salinas, MD, PhD

**********

[NOTE: If reviewer comments were submitted as an attachment file, they will be attached to this email and accessible via the submission site. Please log into your account, locate the manuscript record, and check for the action link "View Attachments". If this link does not appear, there are no attachment files.]

To ensure your figures meet our technical requirements, please review our figure guidelines: https://journals.plos.org/plosone/s/figures

You may also use PLOS’s free figure tool, NAAS, to help you prepare publication quality figures: https://journals.plos.org/plosone/s/figures#loc-tools-for-figure-preparation.

NAAS will assess whether your figures meet our technical requirements by comparing each figure against our figure specifications.

Attachment Submitted filename: Article Review.docx

# Author comment: Author response to Decision Letter 1

**DOI:** 10.1371/journal.pone.0357388.r002

28 Apr 2026

Response to Reviewers: PONE-D-25-68859

Prenatal Substance Exposure and 5-Year Child Protection Outcomes Among Infants Not Reported at Birth

April 21, 2026

Thank you very much for taking the time to thoughtfully review our manuscript submission. We have responded to each of your edits and comments below. We look forward to a review of this revision.

Reviewer Comment Author Response Location of Revision

1. Please ensure that your manuscript meets PLOS ONE's style requirements, including those for file naming. The PLOS ONE style templates can be found at

https://journals.plos.org/plosone/s/file?id=wjVg/PLOSOne_formatting_sample_main_body.pdf and https://journals.plos.org/plosone/s/file?id=ba62/PLOSOne_formatting_sample_title_authors_affiliations.pdf

Thank you – we have reviewed these templates and ensured that our manuscript complies with the style requirements. Throughout (revisions to Title Page)

2. Thank you for stating in your Funding Statement:

[JR was supported by the National Institute on Drug Abuse of the National Institutes of Health under award number F31DA057756.The NIH played no role in the study design, data collection, analysis, preparation, nor decision to publish this manuscript.

RR was supported by the Centers for Disease Control and Prevention, under award number K01CE003548. The CDC played no role in the study design, data collection, analysis, preparation, nor decision to publish this manuscript.

LF was supported by the National Institute of Mental Health of the National Institutes of Health under award number R01MH138475.The NIH played no role in the study design, data collection, analysis, preparation, nor decision to publish this manuscript.].

Please provide an amended statement that declares *all* the funding or sources of support (whether external or internal to your organization) received during this study, as detailed online in our guide for authors at http://journals.plos.org/plosone/s/submit-now. Please also include the statement “There was no additional external funding received for this study.” in your updated Funding Statement.

Please include your amended Funding Statement within your cover letter. We will change the online submission form on your behalf.

Thank you; we have amended the funding statement and relocated it to the cover letter. COVER LETTER

3. We note that you have indicated that there are restrictions to data sharing for this study. For studies involving human research participant data or other sensitive data, we encourage authors to share de-identified or anonymized data. However, when data cannot be publicly shared for ethical reasons, we allow authors to make their data sets available upon request. For information on unacceptable data access restrictions, please see http://journals.plos.org/plosone/s/data-availability#loc-unacceptable-data-access-restrictions.

Before we proceed with your manuscript, please address the following prompts:

a) If there are ethical or legal restrictions on sharing a de-identified data set, please explain them in detail (e.g., data contain potentially identifying or sensitive patient information, data are owned by a third-party organization, etc.) and who has imposed them (e.g., a Research Ethics Committee or Institutional Review Board, etc.). Please also provide contact information for a data access committee, ethics committee, or other institutional body to which data requests may be sent.

b) If there are no restrictions, please upload the minimal anonymized data set necessary to replicate your study findings to a stable, public repository and provide us with the relevant URLs, DOIs, or accession numbers. Please see http://www.bmj.com/content/340/bmj.c181.long for guidelines on how to de-identify and prepare clinical data for publication. For a list of recommended repositories, please see https://journals.plos.org/plosone/s/recommended-repositories. You also have the option of uploading the data as Supporting Information files, but we would recommend depositing data directly to a data repository if possible.

Please update your Data Availability statement in the submission form accordingly.

Thank you. Yes, the data are held by public agencies and permitted for use through restrictive data use agreements that are not transferrable. We have amended the data availability statement and included the contact information of the ethics review body. SUBMISSION FORM and manuscript Line 84

4. Please ensure that you include a title page within your main document. You should list all authors and all affiliations as per our author instructions and clearly indicate the corresponding author. Thank you – we have moved our title page to the manuscript file. LINES 1 – 23

5. Please include your full ethics statement in the ‘Methods’ section of your manuscript file. In your statement, please include the full name of the IRB or ethics committee who approved or waived your study, as well as whether or not you obtained informed written or verbal consent. If consent was waived for your study, please include this information in your statement as well. Thank you – we have added this information to the manuscript. LINES 81-87

Reviewer 1: P205 line 220, there is a space between 2.2_ ), and P234 line 252 “populations” is misspelled Thank you for these edits; changes have been made. LINE 246; LINE 289

Reviewer 2:

Title and abstract: The authors should indicate the study design (retrospective cohort study) either in the abstract or in the title. Currently, the abstract only indicates “cohort analysis”, which is insufficient. Thank you; we have added this language to the abstract, methods, and discussion sections. LINE 28

Methods: Indicate the study design as well at the beginning of the methods section in compliance with STROBE guidelines for observational studies (Best practice: https://www.strobe-statement.org/). Since the study is a cohort study that uses data already collected in the past, the design is a retrospective cohort study. Yes, we have added this language LINES 98 & 267

Although authors cited a publication with the details of the record linkage, but a very brief description is still needed. For instance, it is necessary they declare that it was a probabilistic record linkage using machine learning rather than deterministic and also they can report the accuracy of the linkage. The cited publication compares three methods (three record linkage solutions: PRLF, ChoiceMaker, and Link Plus) and the reader is uncertain as to which of those was the one used in this study. Thank you – this was added LINES 78 - 82

Results: In paragraph 1 of the results section, the authors stated: “A maltreatment harm report occurred in 0.5% of all births by 6 months, 0.9% by one year, 1.6% by 2 years, and 3.5% by five years. Among IPSE, 2.7% experienced a report of maltreatment harm at 6 months, 4.5% at 1 year, 6.7% at 2 years, and 13.2% at 5 years.” Table 1 was cited as the source, but Table 1 presents percentages for cannabis and other substances instead of all IPSE. Correct the issue in the table 1 by adding a column for total IPSE, or change the narrative to match what is the table (e.g., by comparing “not substance exposed” to “cannabis” and “other substances” group. Ah – thank you. I have recreated the table with an added “any substance exposure” column. LINE 189

Analysis: The techniques used were described (Chi-square, Kaplan Meier, Poisson regression). However, a brief description of model diagnostics and goodness of fit indices is needed if available. For instance, for Poisson regression, the methods need to indicate if the following were consulted: dispersion statistic to detect overdispersion; model adequacy measures (such as deviance, Chi-square); if models were compared (AIC, BIC), and Pseudo-R square. Information about robust variance estimators and model adjustment decisions were added to the Analysis section. LINES 145-149

Please indicate how missing values were handled. The handling of missingness was recorded in the Table Notes, and the volume of missingness has been added to the Measures section. LINE 125, 201

Attachment Submitted filename: Response to Reviewers.docx

# Peer review: Decision Letter 1

**DOI:** 10.1371/journal.pone.0357388.r003

**Authors:** Abraham Salinas-Miranda

17 Aug 2026

Prenatal Substance Exposure and 5-Year Child Protection Outcomes Among Infants Not Reported at Birth

PONE-D-25-68859R1

Dear Dr. Reddy,

We’re pleased to inform you that your manuscript has been judged scientifically suitable for publication and will be formally accepted for publication once it meets all outstanding technical requirements.

Within one week, you’ll receive an e-mail detailing the required amendments. When these have been addressed, you’ll receive a formal acceptance letter and your manuscript will be scheduled for publication.

An invoice will be generated when your article is formally accepted. Please note, if your institution has a publishing partnership with PLOS and your article meets the relevant criteria, all or part of your publication costs will be covered. Please make sure your user information is up-to-date by logging into Editorial Manager at Editorial Manager® and clicking the ‘Update My Information' link at the top of the page. For questions related to billing, please contact billing support.

If your institution or institutions have a press office, please notify them about your upcoming paper to help maximize its impact. If they’ll be preparing press materials, please inform our press team as soon as possible -- no later than 48 hours after receiving the formal acceptance. Your manuscript will remain under strict press embargo until 2 pm Eastern Time on the date of publication. For more information, please contact onepress@plos.org.

Kind regards,

Abraham Salinas-Miranda, MD, PhD

Academic Editor

PLOS One

Additional Editor Comments (optional):

One independent reviewer and academic editor reviewed the paper. There is consensus that all comments have been addressed by the authors, including the elements of study design adequately mentioned, Table 1 corrections, model diagnostics, and handling missing data. Thank you.

Reviewers' comments:

Reviewer's Responses to Questions

**Comments to the Author**

Reviewer #1: All comments have been addressed

Reviewer #2: All comments have been addressed

**********

2. Is the manuscript technically sound, and do the data support the conclusions?

Reviewer #1: Yes

Reviewer #2: Yes

**********

3. Has the statistical analysis been performed appropriately and rigorously?

Reviewer #1: Yes

Reviewer #2: Yes

**********

4. Have the authors made all data underlying the findings in their manuscript fully available?

The PLOS Data policy

Reviewer #1: Yes

Reviewer #2: Yes

**********

5. Is the manuscript presented in an intelligible fashion and written in standard English?

Reviewer #1: Yes

Reviewer #2: Yes

**********

Reviewer #1: Thank you for addressing reviewer comments. This paper makes an important contribution to the literature and informs policy and practice.

Reviewer #2: All comments have been addressed including the elements of study design adequately mentioned, Table 1 corrections, model diagnostics, and handling missing data.

**********

what does this mean?). If published, this will include your full peer review and any attached files.

If you choose “no”, your identity will remain anonymous but your review may still be made public.

**Do you want your identity to be public for this peer review?**  For information about this choice, including consent withdrawal, please see our Privacy Policy

Reviewer #1: **Yes:**  Jennifer Marshall

Reviewer #2: **Yes:**  Abraham A. Salinas-Miranda, MD, PhD

**********

# Editor report: Acceptance letter

**DOI:** 10.1371/journal.pone.0357388.r004

**Authors:** Abraham Salinas-Miranda

PONE-D-25-68859R1

PLOS One

Dear Dr. Reddy,

I'm pleased to inform you that your manuscript has been deemed suitable for publication in PLOS One. Congratulations! Your manuscript is now being handed over to our production team.

At this stage, our production department will prepare your paper for publication. This includes ensuring the following:

* All references, tables, and figures are properly cited

* All relevant supporting information is included in the manuscript submission,

* There are no issues that prevent the paper from being properly typeset

You will receive further instructions from the production team, including instructions on how to review your proof when it is ready. Please keep in mind that we are working through a large volume of accepted articles, so please give us a few days to review your paper and let you know the next and final steps.

Lastly, if your institution or institutions have a press office, please let them know about your upcoming paper now to help maximize its impact. If they'll be preparing press materials, please inform our press team within the next 48 hours. Your manuscript will remain under strict press embargo until 2 pm Eastern Time on the date of publication. For more information, please contact onepress@plos.org.

You will receive an invoice from PLOS for your publication fee after your manuscript has reached the completed accept phase. If you receive an email requesting payment before acceptance or for any other service, this may be a phishing scheme. Learn how to identify phishing emails and protect your accounts at https://explore.plos.org/phishing.

If we can help with anything else, please email us at customercare@plos.org.

Thank you for submitting your work to PLOS One and supporting open access.

Kind regards,

PLOS ONE Editorial Office Staff

on behalf of

Dr. Abraham Salinas-Miranda

Academic Editor

PLOS One
